# Spectral enhancement of PlanetScope using Sentinel-2 images to estimate soybean yield and seed composition

**DOI:** 10.1038/s41598-024-63650-3

**Published:** 2024-07-01

**Authors:** Supria Sarkar, Vasit Sagan, Sourav Bhadra, Felix B. Fritschi

**Affiliations:** 1 Taylor Geospatial Institute, Saint Louis, MO 63108 USA; 2 Department of Earth, Environmental and Geospatial Sciences, Saint Louis University, Saint Louis, MO 63108 USA; 3 Division of Plant Sciences and Technology, University of Missouri, Columbia, MO 65211 USA

**Keywords:** Artificial intelligence, Computer vision, Geographic information system, Precision agriculture, Plant phenotype, Remote sensing, Environmental sciences, Natural variation in plants

## Abstract

Soybean is an essential crop to fight global food insecurity and is of great economic importance around the world. Along with genetic improvements aimed at boosting yield, soybean seed composition also changed. Since conditions during crop growth and development influences nutrient accumulation in soybean seeds, remote sensing offers a unique opportunity to estimate seed traits from the standing crops. Capturing phenological developments that influence seed composition requires frequent satellite observations at higher spatial and spectral resolutions. This study introduces a novel spectral fusion technique called multiheaded kernel-based spectral fusion (MKSF) that combines the higher spatial resolution of PlanetScope (PS) and spectral bands from Sentinel 2 (S2) satellites. The study also focuses on using the additional spectral bands and different statistical machine learning models to estimate seed traits, e.g., protein, oil, sucrose, starch, ash, fiber, and yield. The MKSF was trained using PS and S2 image pairs from different growth stages and predicted the potential VNIR1 (705 nm), VNIR2 (740 nm), VNIR3 (783 nm), SWIR1 (1610 nm), and SWIR2 (2190 nm) bands from the PS images. Our results indicate that VNIR3 prediction performance was the highest followed by VNIR2, VNIR1, SWIR1, and SWIR2. Among the seed traits, sucrose yielded the highest predictive performance with RFR model. Finally, the feature importance analysis revealed the importance of MKSF-generated vegetation indices from fused images.

## Introduction

Soybeans is often referred to as "versatile legume of opportunity" as it contributes to the economic value to a range of industries, while also playing a pivotal role in global food security^1^. Sustainable soybean farming has two folded main goals: the quality and the quantity of the soybean seeds. Quality refers to the nutrients inside the soybean seeds, such as protein, oil, carbohydrate, fiber etc., whereas, the quantity measures the total harvested soybean per acre^2^. Both factors are relevant for the profit and the long-term sustainability of soybean production. In 2021, the global soybean production amounted to approximately 355 million metric tons, with the United States, Brazil, and Argentina as major producers, which encompassing around 80% of the world's production^3^. The total economic impact on the U.S. from the soybean sector averaged $115.8 billion per year^4^. With the growing demand by the expanding food industry, soybean serves as a vital ingredient in various products^5^.

Soybean yield, and to a lesser extent seed composition are important for farmeres when making effective agronomic decisions^6^. The modern technology, genetical modification and precision farming have strongly influence farming industries. These innovations have led to better crop management, enhanced cultivation practices and increased yields^7^. Recent studies show that even though the yield has been on the rise but there have been questions about the seed quality. The soybean seed composition has changed as yield have improved.^8^. This brings attention to the need for accurate information about seed composition to ensure that minimum standards are met. Proper understanding of these attributes allows farmers to select appropriate soybean varieties, allocate resources wisely, and cater to specific end-use demands which contributes to more informed and sustainable farming practices.

Remote sensing and machine learning have been helping digital agriculture for the last few decades^9^. Recent advancements in technologies, big data, and computational efficiency possessed positive impact on crop yields and seed quality estimation^10^. Remote sensing technology can be used to create detailed maps of crop health, biomass estimation^11^, leaf area index, canopy nutrient status etc. which can help farmers to make decisions about planting, fertilizing, and harvesting^12^. Machine learning, which is a subset of artificial intelligence that can be used to analyze the data collected by remote sensing and provide informed decisions^13^. This can help growers and breeders to identify the optimal time for planting, fertilizing, and harvesting, by analyzing weather patterns and other parameters, to optimize crop yields and reduce costs^14^.

Publicly available Sentinel-2 (S2) satellite remote sensing data has comparatively high spectral and temporal resolution designed to provide detailed information about the Earth's land surfaces and coastal zones^15^. Researchers have been using this satellite for crop health monitoring^16^, yield estimation^17^, drought stress assessment^18^, precision agriculture^19^, crop type classification^20^, pest and disease detection^21^, biomass estimation^22^ etc., due to its availability of several near-infrared (NIR) and short-wave infrared (SWIR) bands. However, S2 does not have a very high spatial resolution (~ 10 to 20 m), which can limit its ability to detect small changes in vegetation within field or plot-level. On the other hand, PlanetScope (PS) is a constellation of small dove satellites, which are designed to capture daily images of the earth surface at comparatively better spatial resolution^23^. This process allows the collection of data at frequent intervals which gives detailed information of land cover and land use change. The images have 3 m spatial resolution and 4 spectral bands (blue, green, red, NIR) which helps in detailed mapping of land cover and land use^24^, agricultural monitoring^25^, vegetation changes^26^, and natural resource management^27^ etc. Therefore, while S2 provides more spectral bands but lower spatial resolution, PS has higher spatial resolution with fewer bands.

Image data fusion techniques play a crucial role in combining information from different sensors and platforms to enhance the quality, resolution, and interpretability^28^. There are different types of data fusion technology including traditional methods, deep learning-based methods, and generative adversarial networks (GANs). Traditional methods for multispectral image fusion focus on mathematical and statistical techniques to combine information. Statistical data fusion techniques generalize the input data which makes it hard to get precise information for plot level studies. For example, Intensity-Hue-Saturation (IHS) transformation combines high-resolution panchromatic imagery with lower-resolution multispectral imagery^29^, Principal Component Analysis (PCA) reduces the dimensionality by extracting orthogonal components that capture the most variance^30^, Brovey transform is a linear method that enhances the spatial details of multispectral images by sharpening them using panchromatic imagery^31^. Deep learning techniques for image data fusion have shown promising results in generating high-quality, high-resolution data. However, such methods like GANs are computationally very expensive, artifacts noisy images, deprioritize features from the different data sources, and are ideal for fusing regular camera images rather than earth observation datasets^32^. Simple deep learning-based methods for data fusion within the scope of remote sensing and digital agriculture have the potentiality to overcome computational, interpretability, and optimization challenges.

Several studies showed that remotely sensed images, deep learning, machine learning and data fusion combinedly can address different agricultural issues. These fusion approaches have been used to monitor land use and land cover^33^, assessing within field corn and soybean yield variability^34^, water management^35^, land management and disaster risk assessment^36^. PS images lack a wide range of visible near infrared (VNIR) and short-wave infrared (SWIR) bands. However, S2 can compensate for that. So, fusing these two sensors will give all the valuable properties from both images without losing any information. Few studies highlighted that VNIR and SWIR region are important to understand plant growth stages as it can capture variability in water availability^37^. Leveraging different-resolution imagery from both PS and S2 satellites, our study aims to fill a crucial gap in the existing literature by being the first to deploy kernel-based deep neural networks for estimating soybean seed composition. This multi-sensor data fusion approach not only enriches the spatial and spectral data but also introduces an innovative methodology to agricultural research. The objective of this study is three fold (1) introduce a kernel based fusion approach for combining S2 and PS images of varying spatial and spectral resolutions. (2) Estimate seed traits and yield using newly fused images from various growth stages while the plants remain in the field and to determine which growth stage can predict which seed traits (3) to access the contribution of remotely sensed VNIR and SWIR bands in seed composition analysis. This study's contribution lies in pioneering a novel fusion method to integrate satellite images from two modalities, enabling rich and continuous spectral information about crop canopies at different growth stages.

## Study area and datasets

### Experimental design

**Figure 1:**
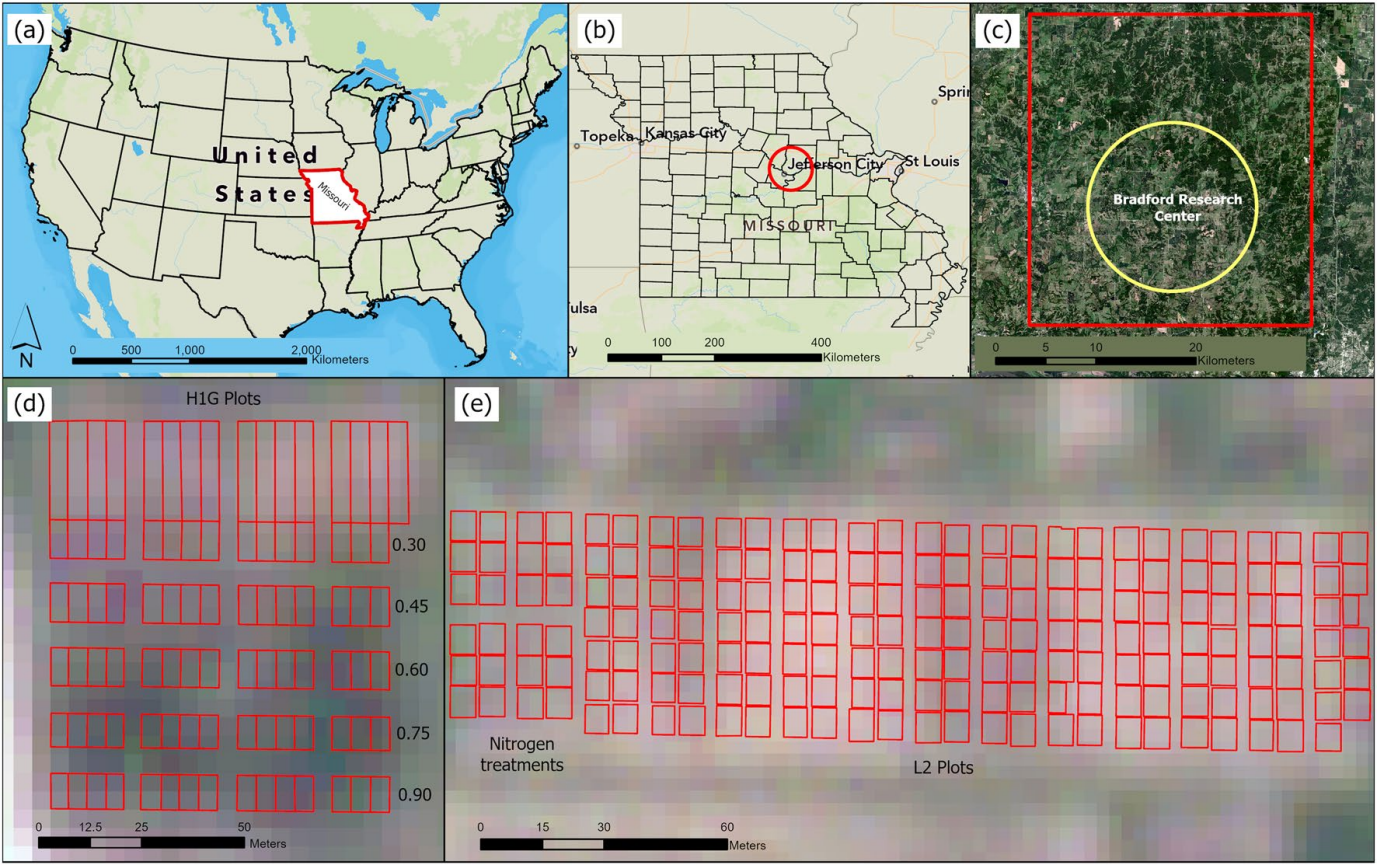
Location of the study area and experimental sites. (**a**) location of the study area in a large perspective; (**b**) Bradford Research Center in Missouri; (**c**) The whole study site in PS image; (**d**) close view of the experimental field H1G with 91 plots; (**e**) zoomed in view of L2 experimental field with 191 plots.

Experiments were conducted at the Bradford Research Center at University of Missouri, Columbia in 2017, 2020 and 2021 (Fig. 1) to investigate the performance of soybean cultivars under different row spacing, fertilization, and rooting depth treatments. All the permission was obtained for the execution of the field experiments. The study site had a humid continental climate, and the growing season temperatures ranged from 13.8 to 25.0 °C with precipitation ranging from 2.53 to 4.46 cm. The first field named H1G contained 91 plots of varying size with five different rooting depth treatments ranging from 0.3 to 0.9 m^38^. The field size was 77 m × 65 m and the plot size was 6.2 m × 3.1 m. Soybean seeds were sown in either 38 cm or 76 cm row spacing with rows sown perpendicular to the rooting depth treatments. The second field named L2 had 191 plots of 6.1 × 6.1 m plot size with a soybean seed row spacing of 38 cm or 76 cm. This field was 228 m long and 61 m width. The fields were planted with different soybean cultivars and weeds were controlled using pre-emergence herbicide applications as well as manual hoeing. The experiments were rainfed and consisted of three varieties ('Pana', 'Dwight', and 'AG3432') and other non-GMO genotypes, and some plots with splits based on the 38 cm and 76 cm row widths.

### Data acquisition

#### Satellite images

The satellite images utilized in this study had two major scenarios, i.e., one where a pair of Sentinel-2 (S2) and PlanetScope (PS) images were collected for developing the fusion model, and another one where only the PS images were downloaded for the specific fields and years. Since in the first scenario, a generalized fusion model must be developed to generate S2-like bands for PS, a significant portion of the Bradford Research Center has been used as the training dataset. The Bradford Research Center includes several agricultural fields where numerous crops are being planted for different experiments. Therefore, the fusion network could take advantage of learning from a diverse set of crops, genotypes, and management conditions. Three image pairs from both S2 and PS were downloaded from the months of July, August, and September. The reason behind choosing these three months was to capture the important vegetative and reproductive stages of different crops in these months.

In the second scenario, PS images were downloaded where frequent image observations for the specific experimental fields and years were needed. The images were analytics ready surface reflectance products, which were stitched together when there were multiple image paths to cover a single S2 image area. For each year, 21 images were downloaded with a 5-day interval starting from roughly 15 days after sowing (DAS). The reason behind choosing DAS as the reference was to normalize the yearly effect and consider the modeling of multiple samples from different years. In addition, several research has indicated that the nutrient status during vegetative growth can influence seed composition which was the main reason behind starting from 15 DAS. Finally, a 5-day interval was considered as the basis of image download frequency to capture crop data at relatively high temporal resolution cloud and haze free days. A total of 84 PS scenes were downloaded for the seed composition estimation scenario.

#### Field data collection

Soybean were harvested at maturity using a small-plot combine to determine seed yield, and to collect a subsample of seeds for seed composition analyses. All the plant seed collections and uses were in accordance with all the national guidelines. The corresponding author of this paper Vasit Sagan undertook the formal identification of the plant material, and no voucher specimen of this material has been deposited in any herbarium. A total of 250 gm harvested seeds were analyzed for seed composition using a DA 7250 NIR analyzer, which is a third-generation diode array NIRS instrument specifically designed for the food and agricultural industries by PerkinElmer. The scanning process involved placing the seeds in a sample dish and using down view reflection or transflectance to detect wavelengths ranging from 900 to 1700 nm. The instrument provided 41 seed composition parameters as percentages. However only protein, oil, sucrose, starch, fiber, and ash were selected as those are of major. For yield the soybean seeds were harvested from each plot and then measured and normalized to $\text{kg ha}^{-1}$. These selected parameters were considered as the ground truth data for the prediction. The datasets generated during this study are not publicly available due to the arrangement with the funding agency.

## Methods

**Figure 2:**
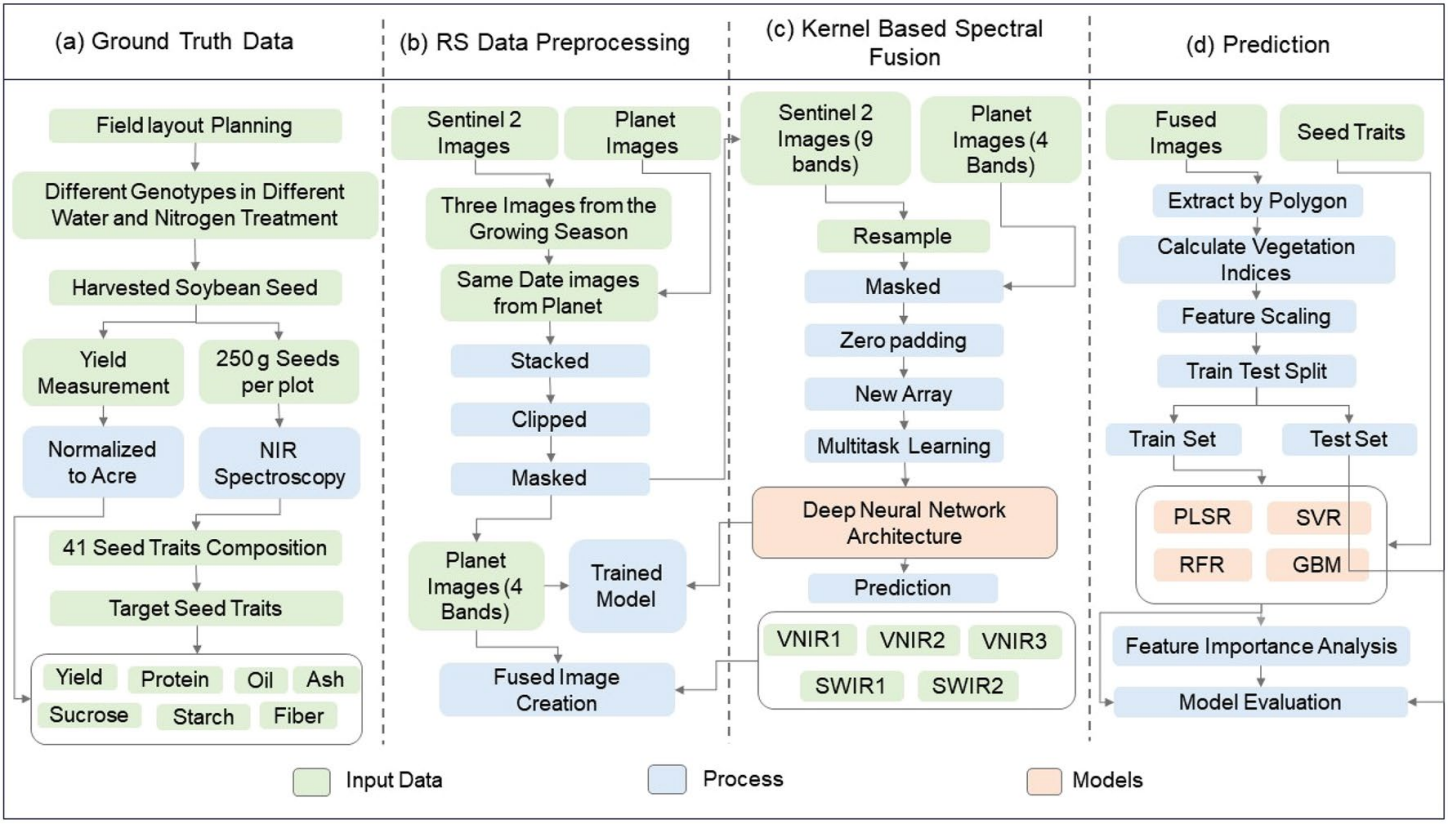
The overall workflow utilized in this study. (**a**) The ground data collection process; (**b**) represents the step-by-step method of satellite data collection processing; (**c**) illustrate summary of the kernel based spectral fusion method; and (**d**) the machine learning methods to predict soybean seed traits and yield.

The overall workflow is illustrated in Fig. 2 which includes aerial and ground data collection, deep neural network-based data fusion, hand crafted feature extraction, machine learning model implementation. A detailed description of the methods is discussed below.

### Multiheaded kernel-based spectral fusion

**Figure 3:**
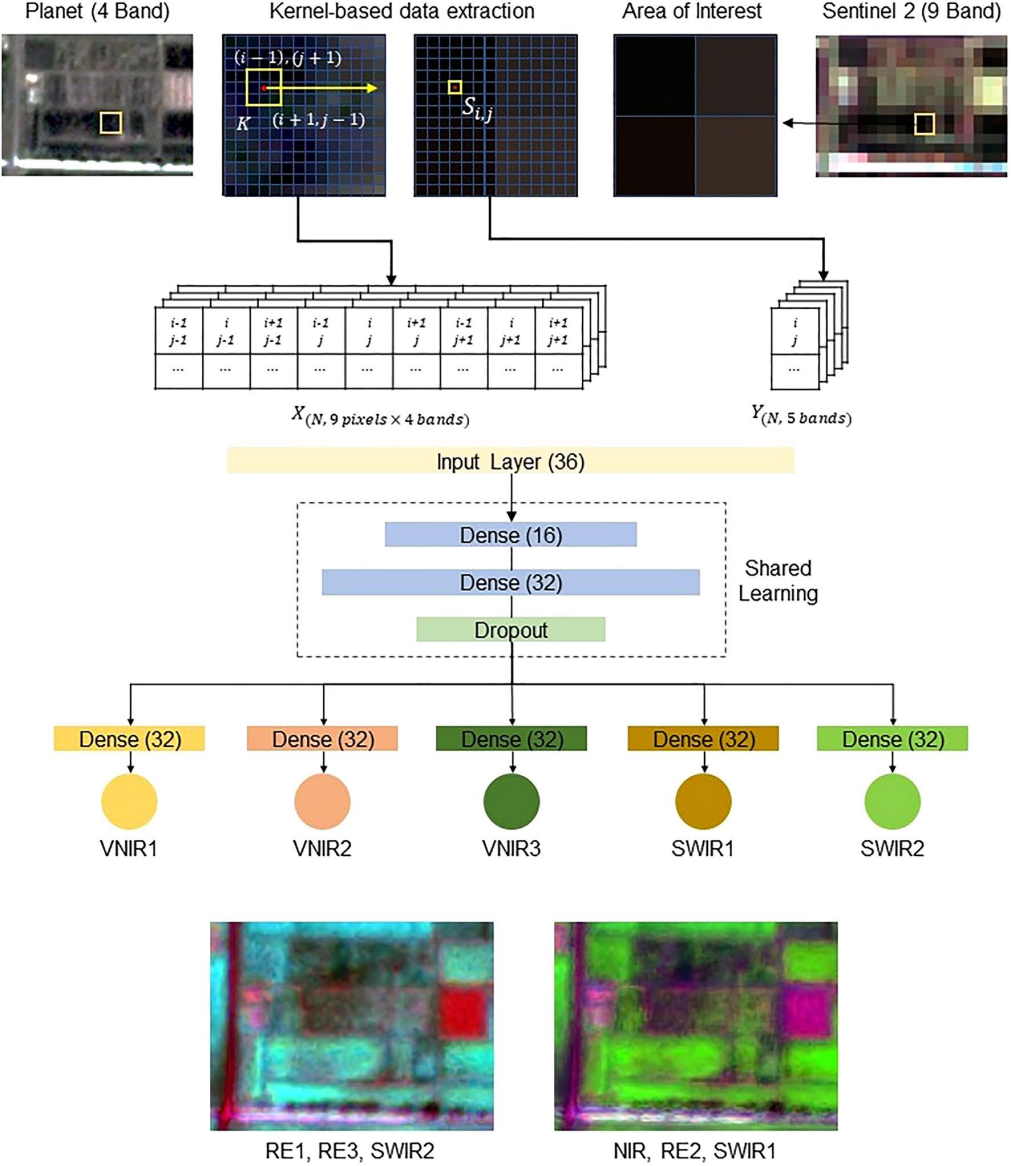
The Kernel Based Spectral fusion in details where it shows how a kernel is moving through an image and predicting a single band, it also shows the overall architecture for each layer, and finally the fused image is shown in false color composite.

The task of the multiheaded kernel-based spectral fusion model was to learn the spatial and spectral characteristics of S2 and PS to enhance the spectral bands of PS while maintaining the spatial details. An overview of the fusion model is illustrated in Fig. 3. The process can be broadly divided into three sections, i.e., kernel-based sample preparation, multi-headed neural network training, and generating the spectral bands for PS.

#### Sample preparation

The principal idea behind kernel-based spectral fusion is to capture the spatial details of the relatively higher resolution image (i.e., PS) during the learning process. The major objective is to develop a complex non-linear model that can capture the relationship between the available bands of PS with the additional bands coming from S2. In this problem, the available bands from PS are the blue (464–517 nm), green (547–585 nm), red (650–682 nm), and near-infrared (846–888 nm), whereas the bands to be predicted from the S2 are VNIR1 (705 nm), VNIR2 (740 nm), VNIR3 (783 nm), SWIR1 (1610 nm), and SWIR2 (2190 nm) which have 20-m spatial resolution. Therefore, both PS and S2 images were first clipped to a matching geospatial shapefile to maintain similar extent. The S2 image bands were then resampled to the matching number of rows and columns from the corresponding PS image to maintain the same number of pixels for both image pairs.

A kernel-based convolutional approach was used to extract salient features from PS and S2 image sets. Utilizing a 3 × 3 matrix kernel $K$ for the $i,j$ coordinates of the resampled S2 image, the corresponding 9 pixels of the PS image were extracted. This operation was repeated for all 4 bands in the PS resulting in 36 values for each pixel in the S2. In addition, the VNIR1, VNIR2, VNIR3, SWIR1, and SWIR2 values from the S2 were also extracted. This process slides the kernel over every pixel, harnessing the kernel's structure to amplify specific image characteristics while suppressing others. The 3 × 3 dimension was chosen for its ability to effectively capture local patterns while maintaining computational efficiency. Finally, two matrices of $X$ and $y$ were generated with shapes of $(273312, 36)$ and $(273312, 5)$, respectively, where 273,312 is the number of pixels. This dataset was used to train the spectral fusion neural network.

Neural Network Architecture.

A multiheaded deep neural network was developed to map out the complex relationship between PS bands and S2 bands. For robust model outputs, the architecture processes a 36-dimensional input vector representing spectral features into a 5-dimensional output vector. The ability to predict multiple outputs in one network allows more generalizability, robustness, and efficiency of the neural network. The architecture model can be divided into two main sections: shared layers and branch-specific layers.

The shared section of the model is constructed with two dense layers, both initialized with the He uniform initializer^39^. The first dense layer maps the 36-dimensional input to a 16-dimensional hidden space with ReLU activation function:

1 $${h}_{1}=RELU({W}_{1}\times X+{b}_{1})$$

Here, ${W}_{1}$ represents the weights of the first dense layer, and ${b}_{1}$ denotes the biases. The output ${h}_{1}$, is then passed through another dense layer to produce a 32-dimensional vector, followed by:

2 $${h}_{2}=RELU({W}_{2}\times {h}_{1}+{b}_{2})$$

To prevent overfitting, a dropout layer with a rate of 0.5 is applied on top of this layer.

After processing through the shared layers, the architecture diverges into multiple branch-specific layers. Each branch represents a different spectral band. For the current design, we have five branches representing VNIR1, VNIR2, VNIR3, SWIR1, and SWIR2. Each branch consists of a dense layer with 32 units followed by another dense layer that outputs a scalar value. The output of the final layer of each branch is transformed by a custom activation function to ensure the output lies between 0 and 1, mimicking the reflectance spectra. Mathematically, the transformation for each branch can be represented as:

3 $${o}_{i}=f({W}_{oi}\times {h}_{2}+{b}_{oi})$$

where, $i$ stands for the branches (i.e., VNIR1, VNIR2, VNIR3, SWIR1, SWIR2), and ${W}_{oi}$ and ${b}_{oi}$ are the weights and biases for the final layer of the $i$ th branch, respectively.

The custom activation function ensures that the outputs of the branches are squeezed between the range^1^, which aligns with the physical constraints of reflectance spectra. This function is defined as:

4 $$f(x)=0.5\times (\text{tanh}\left(x\right)+1)$$

This transformation takes advantage of the $tanh$ function which outputs values between [− 1, 1]. By adding 1, the range becomes^2^, and then multiplying by 0.5 normalizes the range to^1^.

The neural network was trained with the mean squared error loss function. Each branch had a separate loss function of mean squared error. However, the overall network was optimized with the Adam optimizer and the aggregated value of mean squared error loss was used to update the model weights. The learning rate was fixed at 0.0001 with 32 as the batch size. The training dataset was further divided into a separate validation set using an 80%-20% split of the training set. At each epoch, both training loss and validation loss were calculated to see if the model was overfitting or not. We used an early stopping criterion and stopped the model training if validation loss did not decrease for at least 10 epochs.

### Machine learning methods

#### Fused image processing

The final fused images have nine bands ranging from 464 to 2190 nm similar to S2 and have a 3 m spatial resolution like PS images. The fused products had 16-bit integer as GeoTIFF while assembling because PS gives an option to download 16-bit images to be compatible with the S2 sensor. A conversion process was applied to normalize the pixel values within a range of 0–1. At first the plot boundary was created for all the experimental years and fields using ArcGIS Pro software. Subsequently, an automated Python script was employed to extract the average values of all the bands within each plot. To overcome the mixed pixel issue from the adjacent border and plots we took the average values of pixels whose centroids fell entirely within the plot boundaries.

#### Feature extraction

Remote sensing images can capture changes in plant growth stages. However, getting this information can be challenging. Therefore, specialists use specific image bands and features to accurately get the image data. The vegetation features are designed to reduce the interference of outside elements and to properly quantify the plant characteristics. There are several Vegetation Indices (VIs) that can effectively track changes in plant growth during the growing season. In this research, we decided to use 45 different hand-crafted VIs along with the values of nine individual bands for each date for the fused image (details of the features can be found in Appendix A). A total of 945 features were extracted and calculated using an automated python script. We believe adding VIs from VNIR and SWIR bands with the VIs from the PS bands adds more detailed information of plant growth changes. These bands are well known for water content estimation, chlorophyll absorption, soil adjustment, nutrient stress, phenological stages and biomass estimation^40^. By using such a wide range of VIs, we aim to get the most complete and nuanced view of plant features.

#### Machine learning models

Four distinct machine learning models were tested to predict seed composition and yield. At first a simple Partial Least Square Regression (PLSR) method was used. PLSR is a multivariate regression technique that often works better when predictor variables are multicollinear. Here this model was used to extract latent variables that best explain the variance in seed composition and yield in relation to the predictors. Equation 1 represents PLSR where, $Y$ is the mean-centered dependent or target variable, $X$ is the mean centered matrix of independent variables, $\beta$ is the regression coefficient matrix and $\varepsilon$ is the residual matrix.

5 $$Y=X\beta +\varepsilon$$

Secondly, an ensemble learning method named Random Forest Regressor (RFR) was used. This method is based on multiple decision trees at the time of training. For predictions, it takes the average output of individual trees, providing an implicit form of feature selection and averting overfitting. So, the number of trees is the most important factor. The model calculates the feature importance using Mean Squared Error (Eq. 2) where, ${y}_{i}$ is the target, $N$ is the number of instances and $\mu$ is the mean given by $\frac{1}{N}{\sum }_{i=1}^{N}{y}_{i}$. In our study, RFR served as a robust, non-parametric approach to model the nonlinear relationships in the data.

6 $$MSE=\frac{1}{N}{\sum }_{i=1}^{N}{\left({y}_{i}-\mu \right)}^{2}$$

The third method is Gradient Boosting Machine (GBM) which is also a tree-based algorithm. The principal idea behind GBM is that each calculation is done by a simple model and the following calculation is performed to reduce the residual of the last model. The new model is created in the direction of the gradient with reduces residuals^41^. If $({x}_{i},{y}_{i})$ is the given set of data points, where $i=\text{1,2},\dots \dots ,N$, and the loss function of calculating gradient is ${g}_{m}(x)$, the input space is split into disjoint regions ${R}_{1m},{R}_{2m},{R}_{3m},\dots \dots ,{R}_{jm}$, and a constant value is estimated for each region ${b}_{jm}$, where the number of lead nodes per regression tree is $j$. The GBM regression model, $L\left(Y,f\left(x\right)\right)$ can be expressed as:

7 $${g}_{m}\left(x\right)={\sum }_{j=1}^{j}\left({b}_{jm}I\right),x\in {R}_{jm}$$

8 $$I\left(x\in {R}_{jm}\right)=\left\{\begin{array}{c}1,x\in {R}_{jm};\\ 0,other;\end{array}\right.$$

9 $$L\left(Y,f\left(x\right)\right)={\sum }_{i=1}^{n}{\left(Y-f(x)\right)}^{2}$$

The final method is Support Vector Machine (SVR) which performs linear regression in a higher (infinite) dimensional space by mapping input data into that space and determining a hyperplane which best estimates the data. The kernel trick is often employed in SVR, allowing for more complex relationships to be captured. In our context, SVR aimed to discern both linear and non-linear patterns in the seed composition and yield data. The SVM optimization problem can be expressed by introducing the Lagrange function shown in Eq. 3 where, $({x}_{i},{y}_{i})$ is the given set of data points, $i=\text{1,2},\dots \dots ,N$, and ${\alpha }_{i}$ and ${\alpha }_{i}^{*}$ are Lagrange multipliers. Which makes the regression function as Eq. 10.

10 $${\text{max}}\left\{-\frac{1}{2}\sum_{i,j=1}^{N}\left({\alpha }_{i}-{\alpha }_{i}^{*}\right)\left({\alpha }_{i}-{\alpha }_{j}^{*}\right)k\left({x}_{i},{y}_{i}\right)-\varepsilon \sum_{i=1}^{N}\left({\alpha }_{i}+{\alpha }_{i}^{*}\right)+\sum_{i=1}^{N}{y}_{i}\left({\alpha }_{i}+{\alpha }_{i}^{*}\right)\right\}$$

11 $$f\left(x\right)={\sum }_{i=1}^{N}\left({\alpha }_{i}^{*}-{\alpha }_{i}\right)k\left({x}_{i},x\right)+b$$

#### Model training

Our study utilized 54 features encompassing individual spectral bands and a suite of vegetation indices which were captured at each 21 DAS interval. To predict specific seed variables four distinct models were trained on a multiyear dataset of 426 samples. We employed a systematic 70–30 train-test split for model validation to ensure robustness in our evaluation.

Each fused image feature was handled on its own to keep track of the exact phenology of the corresponding growth stage of the plants. Each DAS took its own features and used that information to train a model. A fine hyperparameter tuning was done for each prediction model to make them work better and give more accurate results. For PLSR we took the number of components from 1 to 10. For RFR the number of estimators was 10, 50 and 100 with a maximum depth of 10, 30 and 50. Here the maximum features were 'sqrt', 'log2', 1. The highest number of estimators, learning rate and maximum depth of GBM was 500, 0.1 and 10 respectively. For SVR the gamma was 0.1, 0.5, 1.0 and the c was 0.1, 1.0 while training the model.

#### Model evaluation

Three commonly used model evaluation metrics have been used in this study namely Coefficient of Determination (R-squared), Root Mean Squared Error (RMSE), Normalized Root Mean Squared Error (NRMSE). R-squared (R2) is a statistical metric that represents the percentage of the variance for a dependent variable explained by an independent variable. It describes at what level the variables can be explained to one another calculated as ${R}^{2}$ (Eq. 12) where ${y}_{i}$ is the actual value ${\widehat{y}}_{i}$ is the predicted value, and ${\overline{\overline{y}}}_{i}$ is the mean of actual values. RMSE measures the quality of the fit of the model. In other words, it quantifies how spread out these errors are. RMSE is calculated by computing the differences between predicted and observed values, square each residual, then compute the average of these squared residuals, and finally, take the square root of this average (Eq. 13). NRMSE is a type of RMSE that aims to minimize the scale-dependency when comparing model performance across different units of measurement. It transforms the error metric into a relative scale, typically ranging between 0 and 1. Equation 14 shows the calculation method for NRMSE where ${y}_{i, max}$ and ${y}_{i, min}$ are the maximum and minimum observed values respectively. These metrics are frequently employed in prediction studies in geography, agriculture, earth science, medicine, and environmental research. The equations for the calculation are given below.

12 $${R}^{2}=1-\frac{{\sum }_{i=1}^{n}{\left({y}_{i}-{\widehat{y}}_{i}\right)}^{2}}{{\sum }_{i=1}^{n}{\left({y}_{i}-{\overline{y} }_{i}\right)}^{2}}$$

13 $$RMSE=\sqrt{\frac{{\sum }_{i=1}^{n}{\left({y}_{i}-{\widehat{y}}_{i}\right)}^{2}}{n-1}}$$

14 $$NRMSE=\frac{\sqrt{\frac{{\sum }_{i=1}^{n}{\left({y}_{i}-{\widehat{y}}_{i}\right)}^{2}}{n-1}}}{{y}_{i,max}-{y}_{i,min}}$$

## Results

### Ground data exploration

**Figure 4:**
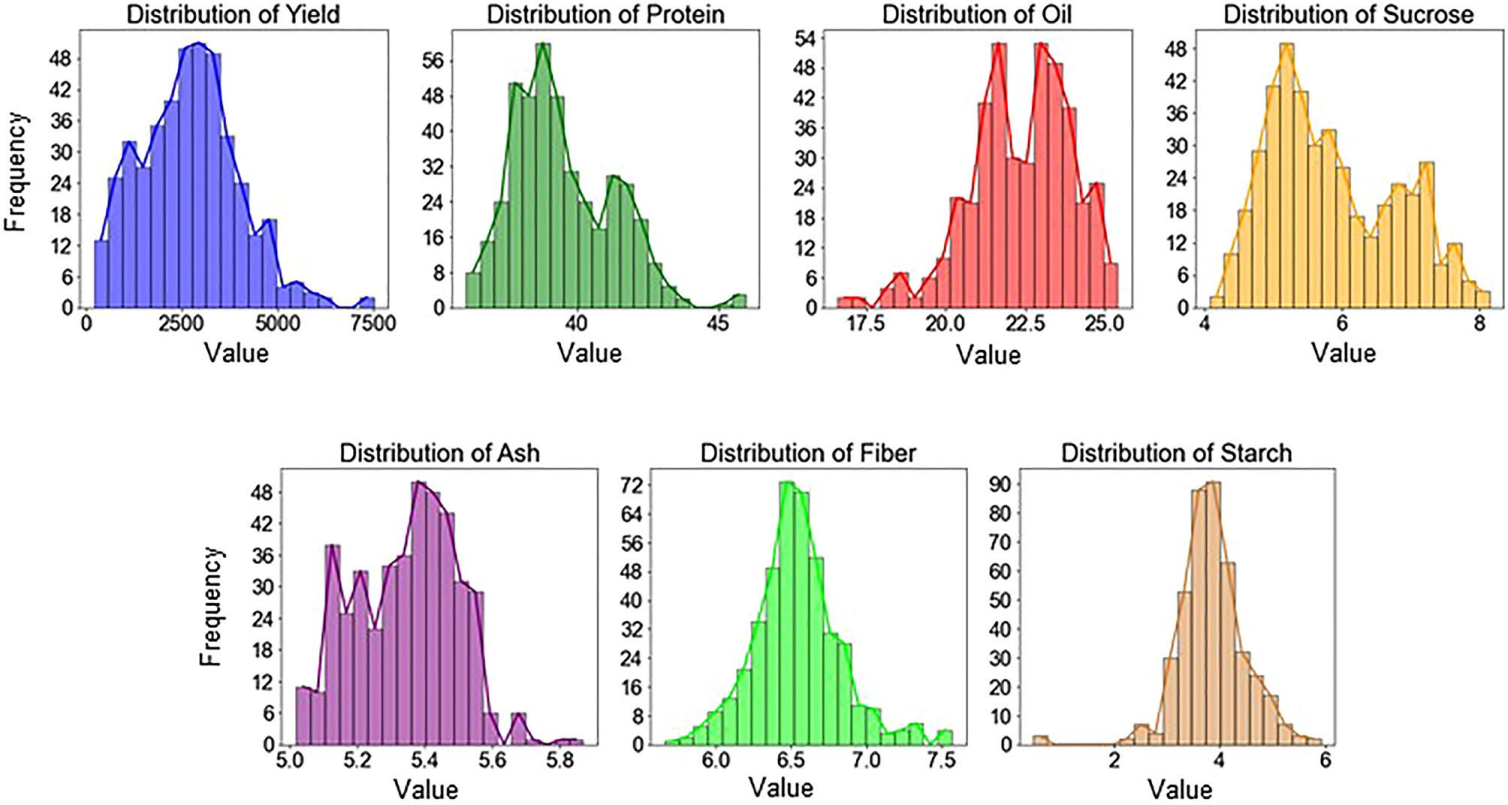
Histogram showing the distribution of each seed traits where the x axis and y axis represents value and frequency respectively.

The seed composition data (in percentage of samples) provides a comprehensive overview of various seed traits found in each seed sample. Figure 4 illustrates a histogram plot for all the seed components. The distribution of protein, oil, sucrose, and ash exhibited a bimodal pattern, while fiber and starch were approximately normally distributed. It also indicates that protein and yield were skewed to the right whereas oil and sucrose were skewed to the left. The statistical analysis (Table 1) of the ground truth data also provided the mean values across years. Variability was present within the data e.g., protein exhibited a standard deviation of 1.76, whereas oil had a slightly lower variability with a standard deviation of 1.60. The sample's minimum and maximum values further explain the range of data for instance, protein concentrations value ranges from a minimum of 36.1% to a maximum of 45.94%. Whereas oil stays between 16.61% and 25.39%. Also, the interquartile range captured by the 25% and 75% percentiles provides insight into the central spread of the data.

**Table 1:** Descriptive statistics of the explainable variables for samples collected from both fields and all years.

Statistics	Seed composition traits (%)						Yield (kg/ha)
	Protein	Oil	Sucrose	Fiber	Ash	Starch	
Mean	39.49	22.33	5.84	6.54	5.34	3.84	2700.20
Standard deviation	1.76	1.60	0.92	0.29	0.15	0.64	1271.81
Minimum	36.1	16.61	4.06	5.66	5.02	0.48	211.64
25% Percentile	38.15	21.29	5.11	6.36	5.22	3.49	1786.19
50% Percentile	39.10	22.52	5.62	6.52	5.36	3.81	2692.17
75% Percentile	40.86	23.52	6.63	6.7	5.45	4.18	3483.19
Maximum	45.94	25.39	8.15	7.57	5.87	5.91	7515.06

### Performance of MKSF and fused image properties

**Figure 5:**
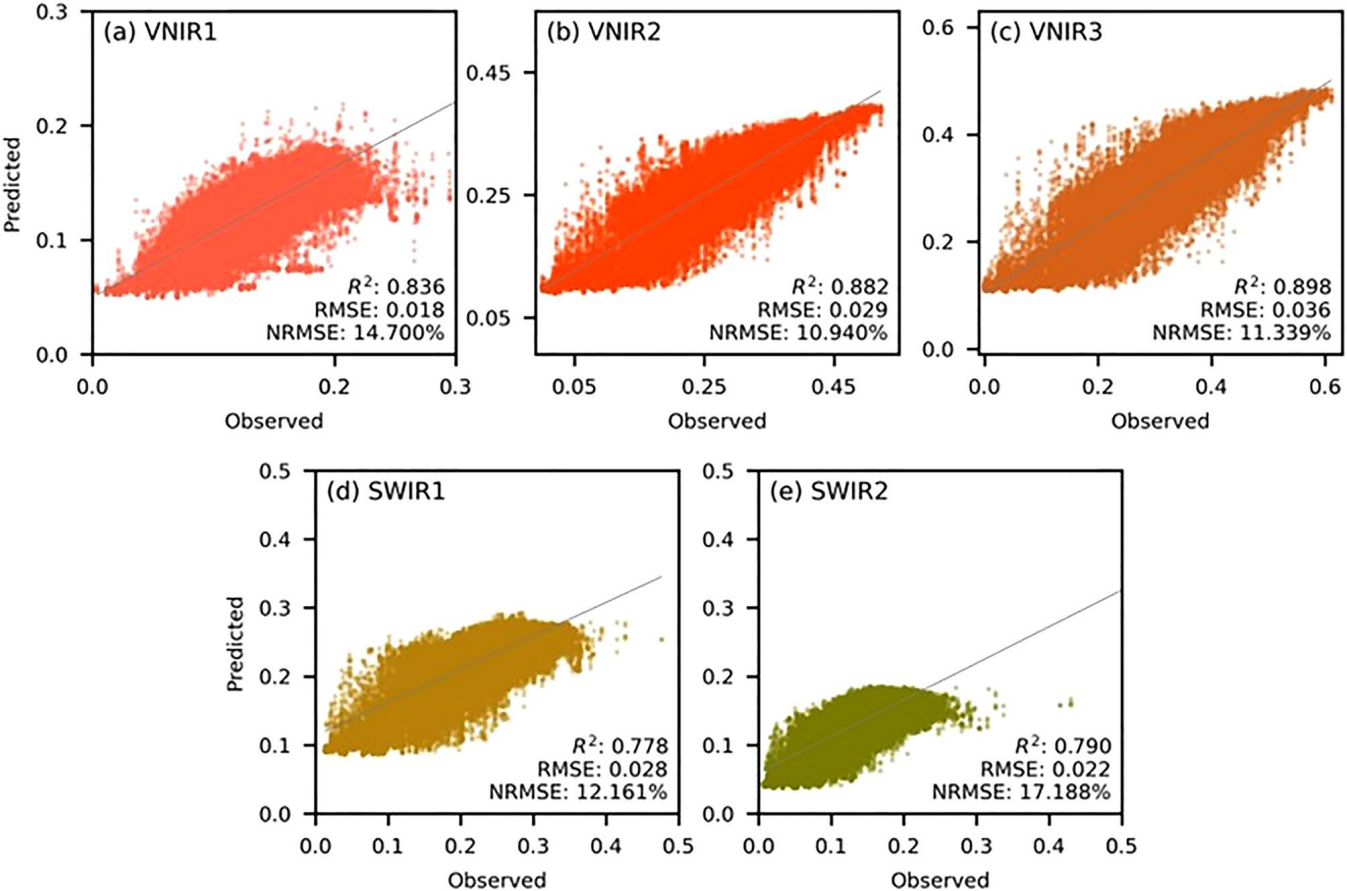
Scatter plot for the predicted bands including VNIR1, VNIR2, VNIR3, SWIR1, SWIR2 with their ${R}^{2}$ and NRMSE. The x-axis represents the observed values, which is the actual pixel values from the test set and the y-axis shows the predicted values.

The overall prediction performance of VNIR1, VNIR2, VNIR3, SWIR1, and SWIR2 using the MKSF network is illustrated in Fig. 5. The results indicate that VNIR3 provided the highest performance (${R}^{2}=0.90$), followed by VNIR2 (${R}^{2}=0.88$), VNIR1 (${R}^{2}=0.84$). The performance of both SWIR1 and SWIR were close to each other (${R}^{2}\approx 0.79$) and showed poorer performance compared to the VNIR bands. In case of the VNIR results, the model seem to underperform on the higher values, specifically for the VNIR1 (Fig. 5a) where observed values (actual pixel values) greater than 0.2 were underpredicted to below 0.2. Similar observation was seen for VNIR2 and VNIR3 but to a smaller extent. However, the model performed the worst for both SWIR1 and SWIR2 where the values higher than the mean were severely underpredicted.

**Figure 6:**
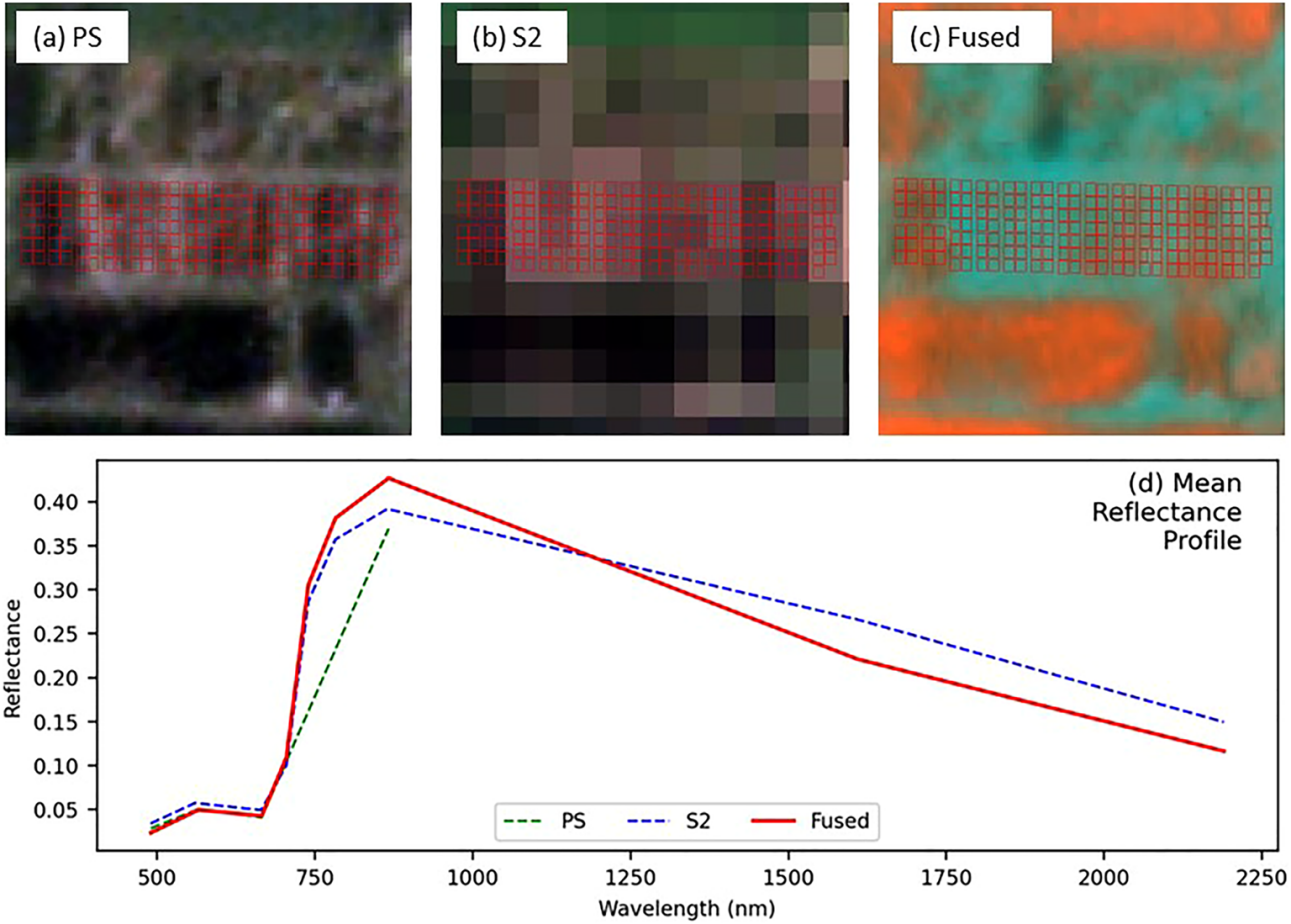
Shows the fused image and its properties. (**a**) Represents the close view of L2 field from PS images; (**b**) illustrating S2 image; (**c**) the fused image in a false color combination; (**d**) the average spectra for the L2 field from both sensor and the fused image where the x-axis represents wavelength and y-axis shows reflectance value.

A spectral profile shows the way light reflects or absorbs across different wavelengths of electromagnetic spectra. Figure 6 shows the spectral profiles for PS, S2, and fused image for the same vegetative pixel. Because of photosynthesis, healthy vegetation tends to absorb light in the blue (band 1) and red (band 3) light. The spectral profile shows the similar dips in all three spectral signatures. Moderate reflectance in the green band has been seen which typically represents healthy vegetation. The cellular structure of the healthy vegetation strongly reflects near-infrared light. The following Fig. 5 shows a strong reflection in NIR band from PS image. However, the reflectance of the S2 and fused were low (around 0.2) but close to each other. High reflectance in the NIR band is a key indicator of healthy vegetation. The predicted 5 bands from S2 images gave reflectance almost similar to the original S2 image. Band 5, 6, and 7 are the red edge bands of the spectrum where the reflectance of vegetation increases rapidly. The high reflectance value in these regions indicates healthy vegetation. The shortwave infrared (SWIR) region is well known for water absorption. Healthy vegetation usually absorbs a significant amount of SWIR light and as a result a drop in SWIR bands is seen. Both the S2 and fused image show a dip, which indicates healthy, well-hydrated vegetation. Overall, the spectral signature of fused image and the S2 image were similar, indicating a valid fused image from the kernel based fusion process.

### Model performance on seed composition estimation

**Figure 7:**
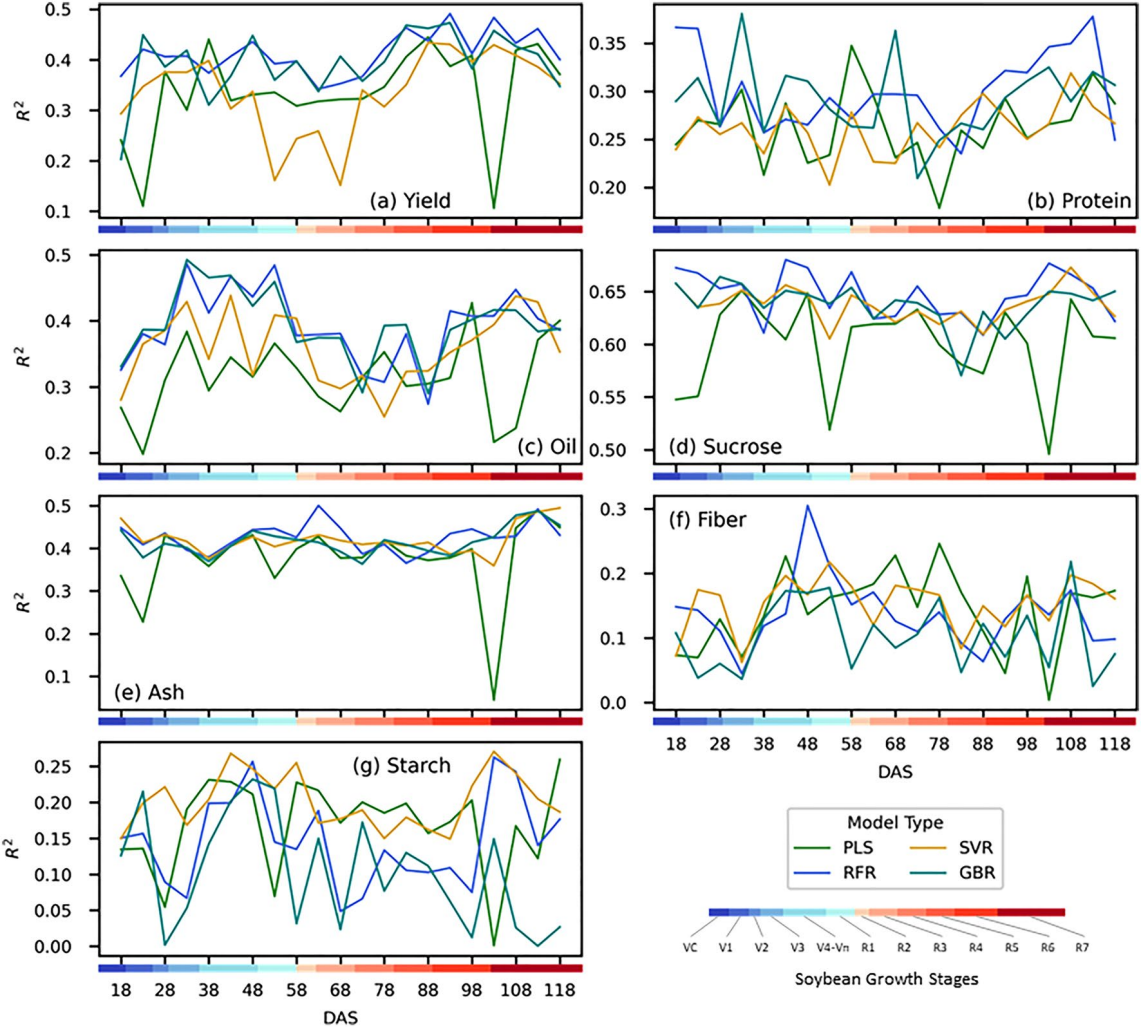
The variation of seed composition prediction based on different growth stages where the x-axis represents the days after sowing (DAS) and the y-axis shows the R-squared. Each rectangle box is for a seed trait and different models are shown in a particular color. The blue to red color bar shows the soybean growth stage.

The comparative analysis of the four machine learning models, i.e., PLSR, SVR, RFR, and GBR, across different seed composition traits revealed varying degrees of success. Among the traits, sucrose (Fig. 7d) demonstrated the most promising results, with the RFR model ($${R}^{2}=0.68)$$ consistently outperforming other models across the DAS range. In terms of most of the traits, RFR tended to outperform all other models and showed consistent behavior for different DAS sets. However, the performance of the models with protein, fiber and starch showed the nonsignificant test set metrics ($${R}^{2}$$ ranging from 0.0 to 0.35).

Yield (Fig. 7a) with the RFR model showed consistent ${R}^{2}$ ranging from 0.36 to 0.49 across the DAS ranges, where the peak was reached at 93 DAS (between R4 to R5 stage). PLSR tend to perform the worst in the initial DAS stages (18 to 33) but reached its peak performance right at 38 DAS (${R}^{2}=0.44$). GBR tended to follow the performance of RFR closely but showed some variability across DAS, and SVR tended to perform poorly with DAS ranging from 53 to 68. Although the overall performance of protein (Fig. 7b) was lower, comparatively better performance was achieved with RFR at the later DAS stages (i.e., 108 to 118 DAS, representing R6 stage). However, the first two DAS stages showed comparative results but varied across different models. The models with oil (Fig. 7c) initially started with lower performance but peaked at the range of 33 and 53 (V3 to R1), while showing a plateau after 53 DAS. A consistently better performance was also achieved from both RFR and GBR (${R}^{2}=0.49$) at 33 DAS. A similar pattern was also observed in case of sucrose (Fig. 7d), where the peak performance (${R}^{2}\approx 0.67$) was achieved by RFR at both 18 DAS (VC to V1 growth stage), 43 DAS (V4 to R1 stage), and 103 DAS (R7 stage). For ash (Fig. 7e), we have seen the lowest model variability as most of the models (except for SVR) showed similar performance (${R}^{2}$ ranging from 0.4 to 0.5) with different DAS groups. Finally, both fiber (Fig. 7f) and starch (Fig. 7g) showed the least ${R}^{2}$ along with high variability across different models and DAS. In summary, while the optimal model and DAS combination varies based on the specific trait, certain patterns emerge. The RFR model's strength for yield and the consistent mid-range DAS peak performance for several traits underscore the significance of a targeted approach for optimal seed composition prediction.

**Figure 8:**
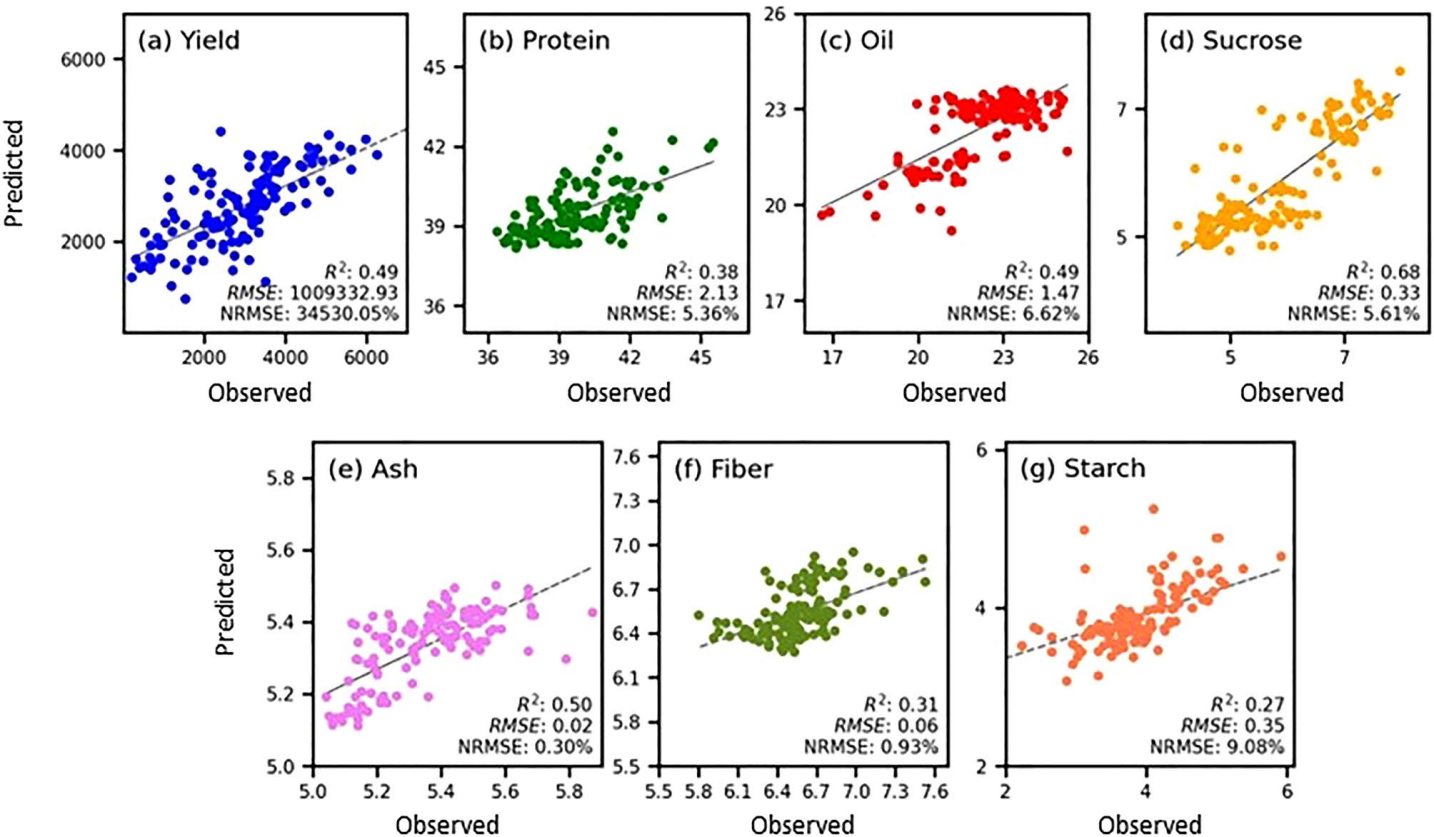
Scatter plot illustrating the total yield and seed composition traits against their top-performing models, where x-axis displays the observed measurements (i.e., actual ground truth values measured using NIR spectroscopy), while the y-axis presents the predicted values.

Figure 8 shows the relationships between the measured and predicted values for the test-set samples of different traits with the best performing model which indicates the models performed well for the observed values that are close to its mean. Models performed relatively better for yield, oil, sucrose, and ash, whereas the over and underestimation of the extreme values led to poor performance metrics for protein, fiber, and starch.

### Feature importance for seed composition traits

**Figure 9:**
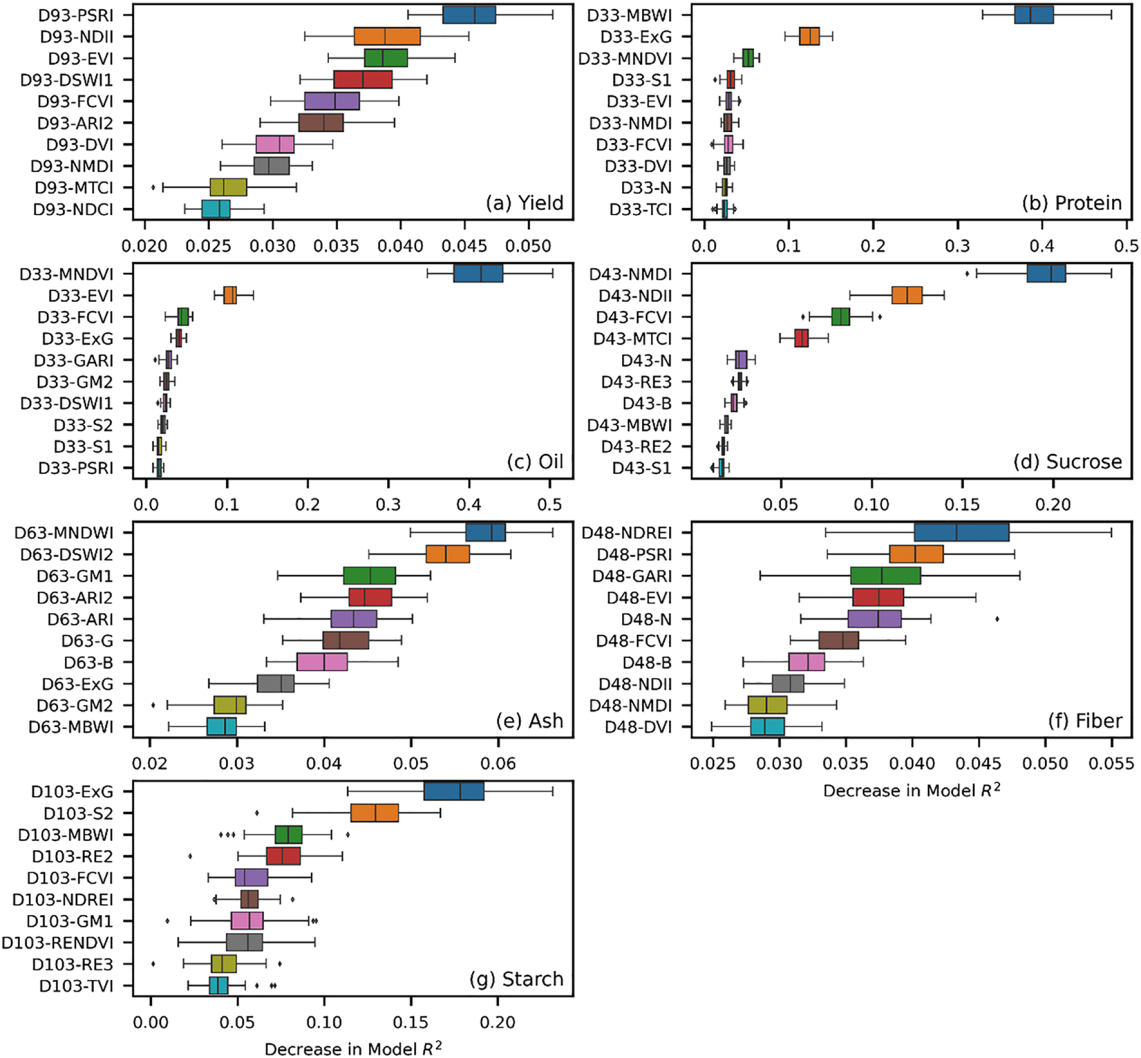
The top 10 most important features for the prediction of each soybean traits and yield. The x-axis represents the decrease in model ${R}^{2}$ whereas the y-axis represents the most important feature contributing to the prediction models.

In this study, a total of 45 features were considered during different modeling paradigms. To understand which feature contributed the most in the modeling performance, we calculated the permutation feature importance score for the best performing model from each seed composition trait. In the permutation feature importance algorithm, the procedure calculates the difference between the model’s baseline metric (with all features unaltered) and its metric with a feature’s values permuted. Therefore, a higher positive permutation feature importance score indicates that permuting the feature’s values decreased the model’s performance. A higher positive value implies the feature is more important for the model’s prediction capability. Figure 9 shows the 10 best performing features for the best models from each trait.

The placement of fused vegetation indices at the top of feature importance plots indicates the importance of having the additional VNIR1, VNIR2, VNIR3, SWIR1 and SWIR2 bands as model inputs. The range of x-axis for different plots indicates how much the model ${R}^{2}$ changes based on permuting certain features. In case of yield (Fig. 9a), most of the features showed slight change in ${R}^{2}$ difference (ranging from 0.02 to 0.05) and the mean value (middle of the whiskers) of each feature did not show drastic difference in each other. However, the two most important features were PSRI (Plant Senescing Reflectance Index) comprised of red, blue, and VNIR2^42^; and NDII (Normalized Difference Infrared Index) composed of NIR, SWIR1, and SWIR2^43^, which includes bands that were the result of fusion. In contrast, the third most important feature was found as EVI (Enhanced Vegetation Index), comprised of NIR, red, and blue bands^44^ that are not part of the fused image. This scenario was observed for other traits as well. This indicates that a combination of vegetation indices coming from both 4 band and fused 9 band images were the key in explaining the yield and seed composition traits. Among the other traits, the top two features for protein (Fig. 9b) and oil (Fig. 9c) showed the considerably greater importance compared to other features as the distance between the first two and others were comparatively higher. For both protein and oil, MBWI (Multi-band Water Index) and MNDVI (Modified Normalized Difference Vegetation Index) were the most important features which is composed of green, red, NIR, SWIR1, and SWIR2^45,46^. For sucrose (Fig. 9d), which was the highest performing trait among others, showed NMDI (Normalized Multi-band Drought Index) and NDII (Normalized Difference Infrared Index) as the most important features, which are both comprised of fused bands^43,47^. NDII was found to be the second most important features for both sucrose and yield. For ash, fiber, and starch, the level of decrease in model ${R}^{2}$ found by the permutation tests was limited (ranging from 0.01 to 0.06). However, the vegetation indices coming from the fused bands were still found on top of the important feature list.

## Discussion

### Imagery based MKSF technique in data fusion

The MKSF neural network presented in this study is a unique approach to the analysis and interpretation of spectral data. Its multi-output architecture is particularly advantageous for tasks that require the simultaneous prediction of multiple spectral bands, as it allows for shared learning^48–50^ across outputs. The use of a custom activation function that bounds the outputs between 0 and 1 is practical for reflectance data and ensures that the model's predictions adhere to physical constraints. We tried with many different linear activation functions, which ended up with poorer performance compared to the results from our custom tanh-based activation function. However, the underprediction of the higher values could be the result of using the custom activation function. Although the underprediction of higher values reduced the overall performance of the model, the bounded nature of the custom activation function provided a natural regularization effect, ensuring the model's predictions remained within the physically plausible range of reflectance.

**Figure 10:**
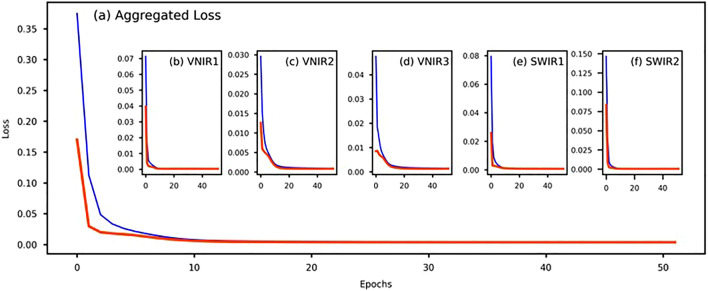
The loss curve while training the deep neural network where number of epochs are in the x-axis and the loss are in the y-axis (**a**) represents the aggregated loss for the total model training; (**b–f**) shows the individual loss curve for each band.

Advantages of this architecture include the efficiency gained from the shared layers, which allow for the reduction of computational resources compared to separate models for each output^51,52^. The use of dropout can also be seen as an effective regularization strategy, potentially leading to better generalization. Figure 10 shows the aggregated loss along with individual loss curve for the bands. The model was trained using an early stopping criterion which stopped the model training if validation loss was not decreasing for at least 10 epochs.

However, there are drawbacks to consider. The shared layers assume that all outputs benefit from the same feature representation, which might not hold if the spectral bands have distinct characteristics requiring specialized feature extraction. In comparison with other spectral fusion techniques such as early fusion, where features are combined at the beginning of the model^53,54^ or late fusion, features are combined during the model training at the end^55,56^, the MKSF finds a middle ground. It allows for both shared representation learning and specialized output-focused adjustments. Traditional fusion techniques might either neglect the benefits of shared learning (as with late fusion) or fail to tailor the model to specific output requirements (as with early fusion).

### Integrated analysis of seed traits and plant phenology

This study demonstrates the effectiveness of using fused images to estimate soybean seed traits and yield. The Random Forest Regression (RFR) model was adept to capture complex interactions between biophysical parameters and spectral signatures^57^-. It showed high efficiency and consistency in yield prediction, especially during critical phenological windows^58^. Interestingly, oil content prediction peaked based on imagery collected between 33 and 53 days after sowing (DAS), which is prior to reproductive growth. Previously, we performed a similar study with unfused PS image-derived vegetation indices for seed composition trait estimation, where the results indicated similar or slightly poorer performance^59^. However, in the future we will set up several experimental sites with varying genotype and environmental combination and then perform a comprehensive analysis with the fused and unfused images.

This study leverages VNIR SWIR band of Sentinel-2 and spatial and temporal resolution of Planet Scope data for their distinct properties. VNIR effectively detects chlorophyll content, indicating plant health and nutrient assimilation capabilities^60^. SWIR responds to water content variations, providing insights into water stress and leaf structural changes^61^. This research added three VNIR bands to the fused image which helped to enhance seed composition and yield estimation. Additionally, incorporating two SWIR bands allowed for comprehensive monitoring of phenological changes and water stress, crucial for understanding plant behavior and improving soybean cultivation strategies.

## Conclusion

Our study demonstrates the feasibility of a multiheaded kernel-based spectral fusion algorithm to enhance the spectral resolution of PS with the help of S2 satellite information. The MKSF combines the usefulness of higher spatial resolution from PS and the better spectral band coverage from S2. We specifically tested the usefulness of having additional VNIR and SWIR bands for estimating soybean seed composition traits to capture the phenological variation of soybean plants. The major findings from our study are:

- The MKSF network successfully enhanced the spectral resolution of PS images. The model showed VNIR3 had the highest predictive accuracy for vegetation health with an ${R}^{2}$ of 0.90, while SWIR bands performed less effectively.
- The sucrose showed the highest predictive performance compared to other seed composition traits (R^2^ ranging from 0.50 to 0.68), followed by oil, ash and yield (R^2^ ranging from 0.50 to 0.66).
- The most optimal growth stage to estimate seed composition varied by trait. Among the better predicted traits, R5 to R6 stages were important for yield, V3 to R1 for oil, R1 for sucrose, R5 to R7 for protein and R4-R5 for ash.
- Among the machine learning models, RFR outperformed other models and showed consistent performance across different traits, followed by the GBR model.
- The permutation-based feature analysis reveals that the most important feature was found from the vegetation index that encompassed at least one of the fused bands derived from the S2 images.

The MKSF holds great promise for future applications in remote sensing and crop phenotyping where comprehensive spectral data are essential. The ability to integrate VNIR and SWIR regions can significantly improve the accuracy of crop trait estimations and can be expanded to other crops across a larger region and for phenotypic traits that require detailed spectral analysis.

## Supplementary Information

Supplementary Information.
