# Supplementary material for: Spectral enhancement of PlanetScope using Sentinel-2 images to estimate soybean yield and seed composition
Source: Sci Rep. 2024 Jul 1;14:15063. doi: 10.1038/s41598-024-63650-3 (PMC11729875; doi:10.1038/s41598-024-63650-3)
Supplement: Supplementary file 1 — Supplementary Information. [file 41598_2024_63650_MOESM1_ESM.docx]

Appendix A: List of features used in the model training

| Vegetation Index | Abbreviation | Equation | Source |
| --- | --- | --- | --- |
| B | Blue Band | $B$ | / |
| G | Green Band | $G$ | / |
| R | Red Band | $R$ | / |
| N | Near Infrared Band | $N$ | / |
| RE1 | RedEdge 1 Band | $RE1$ | / |
| RE2 | RedEdge 2 Band | $RE2$ | / |
| RE3 | RedEdge 3 Band | $RE3$ | / |
| S1 | SWIR 1 Band | $S1$ | / |
| S2 | SWIR 2 Band | $S2$ | / |
| ARI | Anthocyanin Reflectance Index | $(1 / G) - (1 / RE1)$ | Gitelson, Merzlyak [1] |
| ARI2 | Anthocyanin Reflectance Index 2 | $N * ((1 / G) - (1 / RE1))$ | Gitelson, Merzlyak [1] |
| BNDVI | Blue Normalized Difference Vegetation Index | $(N - B)/(N + B)$ | Wang, Huang [2] |
| DSWI1 | Disease-Water Stress Index 1 | $N/S1$ | Apan, Held [3] |
| DSWI2 | Disease-Water Stress Index 2 | $S1/G$ | Apan, Held [3] |
| DSWI3 | Disease-Water Stress Index 3 | $S1/R$ | Apan, Held [3] |
| DVI | Difference Vegetation Index | $N - R$ | Roujean and Breon [4] |
| EVI | Enhanced Vegetation Index | $2.5*(N-R)/(N+6.0*R-7.5*B+L)$ | Huete, Liu [5] |
| EVI2 | Enhanced Vegetation Index 2 | $2.5*(N-R)/(N+2.4*R+L)$ | Jiang, Huete [6] |
| ExG | Excess Green Index | $2 * G - R - B$ | M. Woebbecke, E. Meyer [7] |
| FCVI | Fluorescence Correction Vegetation Index | $N - ((R + G + B)/3.0)$ | Yang, van der Tol [8] |
| GARI | Green Atmospherically Resistant Vegetation Index | $(N-(G-(B-R)))/(N-(G+(B-R)))$ | Gitelson, Kaufman [9] |
| GLI | Green Leaf Index | $(2.0*G-R-B)/(2.0*G+R+B)$ | Louhaichi, Borman [10] |
| GM1 | Gitelson and Merzlyak Index 1 | $RE2/G$ | Gitelson, Kaufman [9] |
| GM2 | Gitelson and Merzlyak Index 2 | $RE2/RE1$ | Gitelson, Kaufman [9] |
| GNDVI | Green Normalized Difference Vegetation Index | $(N - G)/(N + G)$ | Gitelson, Kaufman [9] |
| GRNDVI | Green-Red Normalized Difference Vegetation Index | $(N - (G + R))/(N + (G + R))$ | Wang, Huang [2] |
| MCARI | Modified Chlorophyll Absorption in Reflectance Index | $((RE1-R)-0.2*(RE1-G))*(RE1/R)$ | Daughtry, Walthall [11] |
| MNDVI | Modified Normalized Difference Vegetation Index | $(N - S2)/(N + S2)$ | Jurgens [12] |
| MTCI | MERIS Terrestrial Chlorophyll Index | $(RE2 - RE1) / (RE1 - R)$ | Dash, Curran [13] |
| NDDI | Normalized Difference Drought Index | $\frac{((N-R)/(N+R))-((G-N)/(G+N))}{((N-R)/(N+R))+((G-N)/(G+N)))}$ | Gu, Brown [14] |
| NDII | Normalized Difference Infrared Index | $(N - S1)/(N + S1)$ | Hardisky, Klemas [15] |
| NDREI | Normalized Difference Red Edge Index | $(N - RE1) / (N + RE1)$ | Gitelson and Merzlyak [16] |
| NDVI | Normalized Difference Vegetation Index | $(N - R)/(N + R)$ | Rouse, R H [17] |
| NDYI | Normalized Difference Yellowness Index | $(G - B) / (G + B)$ | Sulik and Long [18] |
| NMDI | Normalized Multi-band Drought Index | $(N - (S1 - S2))/(N + (S1 - S2))$ | Wang and Qu [19] |
| NormG | Normalized Green | $G/(N + G + R)$ | Sripada, Heiniger [20] |
| NormNIR | Normalized NIR | $N/(N + G + R)$ | Sripada, Heiniger [20] |
| NormR | Normalized Red | $R/(N + G + R)$ | Sripada, Heiniger [20] |
| PSRI | Plant Senescing Reflectance Index | $(R - B)/RE2$ | Merzlyak, Gitelson [21] |
| RCC | Red Chromatic Coordinate | $R / (R + G + B)$ | Gillespie, Kahle [22] |
| RENDVI | Red Edge Normalized Difference Vegetation Index | $(RE2 - RE1)/(RE2 + RE1)$ | Gitelson and Merzlyak [23] |
| RGBVI | Red Green Blue Vegetation Index | $(G^{2}-B*R)/(G^{2}+B*R)$ | Bendig, Yu [24] |
| RVI | Ratio Vegetation Index | $N / R$ | Birth and McVey [25] |
| SR | Simple Ratio | $N/R$ | Jordan [26] |
| TCI | Triangular Chlorophyll Index | $\sqrt{1.2*(RE1-G)-1.5*(R-G)*(RE1/R)}$ | Haboudane, Tremblay [27] |
| TRRVI | Transformed Red Range Vegetation Index | $((RE2-R)/(RE2+R))/(((N-R)/(N+R))+1.0)$ | Blanco, Blaya-Ros [28] |
| VARI | Visible Atmospherically Resistant Index | $(G - R) / (G + R - B)$ | [29] |
| ANDWI | Augmented Normalized Difference Water Index | $(B+G+R-N-S1-S2)/(B+G+R+N+S1+S2)$ | Rad, Kreitler [30] |
| MBWI | Multi-Band Water Index | $(2.0* G) - R - N - S1 - S2$ | Wang, Xie [31] |
| MNDWI | Modified Normalized Difference Water Index | $(G - S1) / (G + S1)$ | Xu [32] |
| NDCI | Normalized Difference Chlorophyll Index | $(RE1 - R)/(RE1 + R)$ | Mishra and Mishra [33] |
| TVI | Transformed Vegetation Index | $\sqrt{(((N - R)/(N + R)) + 0.5)}$ | Rouse, R H [17] |
| WDRVI | Wide Dynamic Range Vegetation Index | $(0.1* N - R) / (0.1 * N + R)$ | Gitelson [34] |

1. Gitelson, A.A., M.N. Merzlyak, and O.B. Chivkunova, *Optical properties and nondestructive estimation of anthocyanin content in plant leaves.* Photochemistry and Photobiology, 2001. **74**(1): p. 38-45.

2. Wang, F.-m., et al., *New Vegetation Index and Its Application in Estimating Leaf Area Index of Rice.* Rice Science, 2007. **14**(3): p. 195-203.

3. Apan, A., et al., *Detecting sugarcane 'orange rust' disease using EO-1 Hyperion hyperspectral imagery.* International Journal of Remote Sensing, 2004. **25**(2): p. 489-498.

4. Roujean, J.L. and F.M. Breon, *Estimating Par Absorbed by Vegetation from Bidirectional Reflectance Measurements.* Remote Sensing of Environment, 1995. **51**(3): p. 375-384.

5. Huete, A.R., et al., *A comparison of vegetation indices global set of TM images for EOS-MODIS.* Remote Sensing of Environment, 1997. **59**(3): p. 440-451.

6. Jiang, Z.Y., et al., *Development of a two-band enhanced vegetation index without a blue band.* Remote Sensing of Environment, 2008. **112**(10): p. 3833-3845.

7. M. Woebbecke, D., et al., *Color Indices for Weed Identification Under Various Soil, Residue, and Lighting Conditions.* Transactions of the ASAE, 1995. **38**(1): p. 259-269.

8. Yang, P.Q., et al., *Fluorescence Correction Vegetation Index (FCVI): A physically based reflectance index to separate physiological and non-physiological information in far-red sun-induced chlorophyll fluorescence.* Remote Sensing of Environment, 2020. **240**.

9. Gitelson, A.A., Y.J. Kaufman, and M.N. Merzlyak, *Use of a green channel in remote sensing of global vegetation from EOS-MODIS.* Remote Sensing of Environment, 1996. **58**(3): p. 289-298.

10. Louhaichi, M., M.M. Borman, and D.E. Johnson, *Spatially Located Platform and Aerial Photography for Documentation of Grazing Impacts on Wheat.* Geocarto International, 2001. **16**(1): p. 65-70.

11. Daughtry, C.S.T., et al., *Estimating corn leaf chlorophyll concentration from leaf and canopy reflectance.* Remote Sensing of Environment, 2000. **74**(2): p. 229-239.

12. Jurgens, C., *The modified normalized difference vegetation index (mNDVI) - a new index to determine frost damages in agriculture based on Landsat TM data.* International Journal of Remote Sensing, 1997. **18**(17): p. 3583-3594.

13. Dash, J., et al., *Validating the MERIS Terrestrial Chlorophyll Index (MTCI) with ground chlorophyll content data at MERIS spatial resolution.* International Journal of Remote Sensing, 2010. **31**(20): p. 5513-5532.

14. Gu, Y.X., et al., *A five-year analysis of MODIS NDVI and NDWI for grassland drought assessment over the central Great Plains of the United States.* Geophysical Research Letters, 2007. **34**(6).

15. Hardisky, M., V. Klemas, and a. Smart, *The influence of soil salinity, growth form, and leaf moisture on the spectral radiance of Spartina Alterniflora canopies.* Photogrammetric Engineering and Remote Sensing, 1983. **48**: p. 77-84.

16. Gitelson, A. and M.N. Merzlyak, *Quantitative Estimation of Chlorophyll-a Using Reflectance Spectra - Experiments with Autumn Chestnut and Maple Leaves.* Journal of Photochemistry and Photobiology B-Biology, 1994. **22**(3): p. 247-252.

17. Rouse, J.W.J., et al., *Monitoring Vegetation Systems in the Great Plains with Erts* NASA. Goddard Space Flight Center 3d ERTS-1 Symp, 1974. **1**.

18. Sulik, J.J. and D.S. Long, *Spectral considerations for modeling yield of canola.* Remote Sensing of Environment, 2016. **184**: p. 161-174.

19. Wang, L.L. and J.J. Qu, *NMDI: A normalized multi-band drought index for monitoring soil and vegetation moisture with satellite remote sensing.* Geophysical Research Letters, 2007. **34**(20).

20. Sripada, R.P., et al., *Aerial color infrared photography for determining late-season nitrogen requirements in corn.* Agronomy Journal, 2005. **97**(5): p. 1443-1451.

21. Merzlyak, M.N., et al., *Non-destructive optical detection of pigment changes during leaf senescence and fruit ripening.* Physiologia Plantarum, 1999. **106**(1): p. 135-141.

22. Gillespie, A.R., A.B. Kahle, and R.E. Walker, *Color enhancement of highly correlated images. II. Channel ratio and “chromaticity” transformation techniques.* Remote Sensing of Environment, 1987. **22**(3): p. 343-365.

23. Gitelson, A. and M.N. Merzlyak, *Spectral Reflectance Changes Associated with Autumn Senescence of Aesculus-Hippocastanum L and Acer-Platanoides L Leaves - Spectral Features and Relation to Chlorophyll Estimation.* Journal of Plant Physiology, 1994. **143**(3): p. 286-292.

24. Bendig, J., et al., *Combining UAV-based plant height from crop surface models, visible, and near infrared vegetation indices for biomass monitoring in barley.* International Journal of Applied Earth Observation and Geoinformation, 2015. **39**: p. 79-87.

25. Birth, G.S. and G.R. McVey, *Measuring the Color of Growing Turf with a Reflectance Spectrophotometer1.* Agronomy Journal, 1968. **60**(6): p. 640-643.

26. Jordan, C.F., *Derivation of Leaf-Area Index from Quality of Light on the Forest Floor.* Ecology, 1969. **50**(4): p. 663-666.

27. Haboudane, D., et al., *Remote estimation of crop chlorophyll content using spectral indices derived from hyperspectral data.* Ieee Transactions on Geoscience and Remote Sensing, 2008. **46**(2): p. 423-437.

28. Blanco, V., et al., *Potential of UAS-Based Remote Sensing for Estimating Tree Water Status and Yield in Sweet Cherry Trees.* Remote Sensing, 2020. **12**(15).

29. Gitelson, A.A., et al., *Novel algorithms for remote estimation of vegetation fraction.* Remote Sensing of Environment, 2002. **80**(1): p. 76-87.

30. Rad, A.M., J. Kreitler, and M. Sadegh, *Augmented Normalized Difference Water Index for improved surface water monitoring.* Environmental Modelling & Software, 2021. **140**.

31. Wang, X.B., et al., *A robust Multi-Band Water Index (MBWI) for automated extraction of surface water from Landsat 8 OLI imagery.* International Journal of Applied Earth Observation and Geoinformation, 2018. **68**: p. 73-91.

32. Xu, H.Q., *Modification of normalised difference water index (NDWI) to enhance open water features in remotely sensed imagery.* International Journal of Remote Sensing, 2006. **27**(14): p. 3025-3033.

33. Mishra, S. and D.R. Mishra, *Normalized difference chlorophyll index: A novel model for remote estimation of chlorophyll-a concentration in turbid productive waters.* Remote Sensing of Environment, 2012. **117**: p. 394-406.

34. Gitelson, A.A., *Wide dynamic range vegetation index for remote quantification of biophysical characteristics of vegetation.* Journal of Plant Physiology, 2004. **161**(2): p. 165-173.
